# Studies on anti-inflammatory effect of aqueous extract of leaves of *Holoptelea integrifolia*, Planch. in rats

**DOI:** 10.4103/0253-7613.51348

**Published:** 2009-04

**Authors:** Shrinivas Sharma, K.S. Lakshmi, Arjun Patidar, Abhinav Chaudhary, Sanjay Dhaker

**Affiliations:** Department of Pharmacology, SRM College of Pharmacy, SRM University, SRM Nagar, Kattankulathur - 603 203, Tamil Nadu, India

**Keywords:** Antiinflammatory activity, *Holoptelea integrifolia* edema

## Abstract

**Objectives:**

The purpose of the present study was to investigate the anti-inflammatory properties of aqueous extract of the leaves of *H. integrifolia*, Planch.

**Materials and Methods:**

The hind paw edema was produced in rats by subplanter injection of carageenan. The aqueous extract of *H. integrifolia*, Planch. (AHI) at dose (250 and 500 mg/kg p.o) was given to observe % inhibition of paw edema which were comparable with indomethacin (10 mg/kg p.o) used as a reference drug.

**Results:**

The extract administered orally at doses of 250 and 500 mglkg p.o produced a significant (*P* < 0.05) dose dependent inhibition of edema formation

**Conclusions:**

A significant % inhibition of paw edema by the aqueous extract of leaves of *H. integrifolia*, Planch. and its almost nearby same % inhibition with indomethacin suggest its usefulness as an anti-inflammatory agent.

Acute and chronic inflammatory diseases are still one of the most important health problems in the world. Although several agents are known to treat inflammatory disorders, their prolonged use often leads to gastric intolerance, bone marrow depression, water and salt retention. For this reason there is a need to find and develop new anti-inflammatory drugs with low side effects.[1]

*Holoptelea integrifolia*, Planch. belongs to the family of Urticaceae. It is an important pollen allergen of India and sensitizes almost 10% of the atopic population in Delhi.[2] Some recent explorations have been reported on this plant in which antiviral activity,[3] antioxidant, antimicrobial and wound healing activities[4] are important. Ethnomedically, the leaves and stem bark of this plant were used by local people for skin diseases, obesity,[5] cancer[6] and for wound healing in the form of paste. The fresh material, either stem bark or leaves of the plant, is applied as paste externally twice or thrice a day for wound-healing. The process of wound-healing involves inflammation, cell proliferation and contraction of collagen lattice formation.[7] Hence, the present study was under taken to evaluate the anti-inflammatory activity of aqueous extract of *Holoptelea integrifolia,* Planch. (AHI).

The leaves of *H. integrifolia*, Planch. were collected from the foothills of Yercaud, Tamil Nadu, India. The air-dried and powdered leaves of *H. integrifolia*, Planch. (400 gm) were successfully extracted with petroleum ether by continuous hot percolation method using Soxhlet apparatus, the extract obtained was made free of any solvent by distillation and then aqueous extract was prepared by cold maceration procedure with distilled water.

The experimental protocol was approved by the Institutional Animal Care and Use Committee (IACUC) of the SRM University, Kattankulathur, Tamil Nadu, India. The anti-inflammatory activity was evaluated by the carrageenan-induced paw edema test in the male Wistar strain rats,[8] weighing between 150 and 200 gm. The animals were housed in standard isolation cages (45 × 35 × 25 cm) under environmentally controlled conditions with a 12-h light/12-h dark cycle. Rats were allowed free access to water and standard laboratory rat chow (Hindustan Lever Pvt. Ltd, Mumbai).

Male Wistar rats group I (control) were treated with distilled water (10 ml/kg), used as vehicle. Groups II and III were treated orally with AHI 250 and 500 mg/kg, respectively, while group IV was treated orally with 10 mg/kg of indomethacin used as reference, 30 min before 0.1 ml 1% carrageenan in isotonic saline was injected subplantarly into right hind paws. The contralateral paw was injected with 0.1 ml saline and used as a control. Measurements of paw volume (ml) were made by mercury displacing techniques using plethysmometer. Immediately before and 1, 2, 3 and 4 h after carrageenan injection, percentage increment of paw volume after 1, 2, 3 and 4 h was calculated by Newbould method.[9] The results are expressed as the mean ± S.E. Mean and the statistical significance of differences between groups was analyzed by one-way analysis of variance (ANOVA). *P* < 0.05 was considered as significant.

The subplantar injection of carrageenan caused a time-dependent paw edema in the rat although saline injection caused no swelling (data not shown). In carrageenan-induced paw edema in rats, oral administration of AHI (250 and 500 mg/kg p.o.) inhibited paw swelling dose-dependently at 2, 3, and 4 hr after carrageenan injection (*P* < 0.05) which was comparable with the indomethacin treated group. Percent increment in paw swelling was calculated using the values before carrageenan injection [Figure 1].

**Figure 1:**
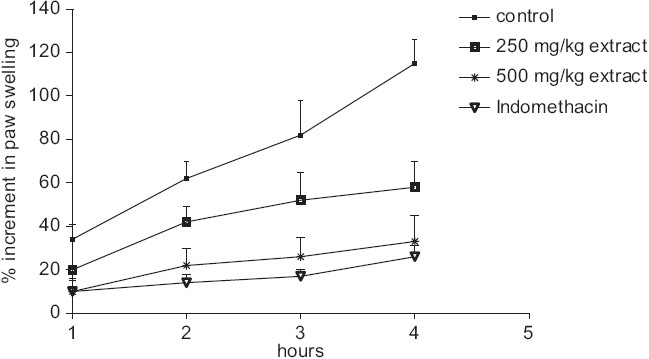
Inhibition of carrageenan-induced paw edema by AHI (250 and 500 mg/kg p.o) in rats. Data are expressed as mean and vertical lines show mean ± S.E (n = 10 for each group). *P < 0.05 versus control

The present data showed that the aqueous extract of leaves of *Holoptelea integrifolia*, Planch. exhibits anti-inflammatory activity. Further studies are in progress in our laboratory to discover which constituent of the extract exerts this activity and to explore its exact mechanism of action.
